# Factors affecting interest in cardiothoracic surgery among junior surgical residents in Nigeria

**DOI:** 10.5830/CVJA-2017-004

**Published:** 2017

**Authors:** Emeka B Kesieme, Umar Abubakar, Olugbenga Olusoji, Ismail Mohammed Inuwa, Kefas John, Ndubuisi Anumenechi

**Affiliations:** Department of Surgery, Irrua Specialist Teaching Hospital, Irrua, Edo State, Nigeria; Department of Surgery, Usmanu Dan Fodio University Teaching Hospital, Sokoto, Sokoto State, Nigeria; Department of Surgery, Lagos University Teaching Hospital, Lagos, Lagos State, Nigeria; Department of Surgery, Aminu Kano University Teaching Hospital, Kano, Kano State, Nigeria; Department of Surgery, Tafawa Belawa University Teaching Hospital, Bauchi, Bauchi State, Nigeria; Department of Surgery, Ahmadu Bello University Teaching Hospital, Zaria, Kaduna State, Nigeria

**Keywords:** open-heart surgery, thoracic surgery, developing countries

## Abstract

**Objective:**

A survey was undertaken to determine the factors that affect interest in cardiothoracic surgery (CTS) among junior surgical residents in Nigeria.

**Methods:**

A cross-sectional study was done using a pilottested, 56-item, semi-structured questionnaire, which was filled in by 238 junior surgical residents in accredited hospitals in Nigeria.

**Results:**

Few of the respondents (8.4%) were committed to specialising in CTS. A minority of them, 28.2 and 2.1%, had assisted in major thoracic procedures and open-heart surgeries, respectively. The relationship between the level of training, rotation in CTS in junior residency and interest in CTS were statistically significant (p < 0.05). The main important factors responsible for the low interest in CTS include the lack of equipment (92%), limited training positions (64.9%), poor or lack of exposure in CTS as a junior resident (63%) and in medical school (58.8%).

**Conclusion:**

There is a dire need to provide facilities and training opportunities to improve the cardiothoracic workforce in Nigeria.

## Introduction

In Nigeria, postgraduate training in surgery is conducted in designated hospitals approved and accredited by the West African College of Surgeons (WACS) and the National Postgraduate Medical College of Nigeria (NPMCN). Junior surgical residents who are interested in cardiothoracic surgery are required to pass Part 1 of the examination of the Faculty of Surgery, WACS or NPMCN in general clinical surgery, after completing a minimum of 24 months’ posting in core surgical specialties.

The training in cardiothoracic surgery lasts for a minimum period of 48 months and involves rotations in general thoracic surgery (24 months), cardiac surgery (12 months), vascular surgery (six months), adult and paediatric cardiology (three months) and cardiopulmonary imaging (three months). There are eight centres partially accredited for cardiothoracic surgery speciality training with approximately 30 positions. These centres do mainly thoracic surgery. In the absence of a viable cardiac surgery centre in the country, cardiac surgery rotation is done in recognised centres in Ghana, South Africa, India and Western Europe.

The number of candidates who sit for the exit/qualifying examination in cardiothoracic surgery of the WACS and NPMCN has remained small. In April 2015, out of the 95 candidates who attempted the exit/qualification examination (Part 2) of the WACS, only four candidates in the entire Anglophone West Africa sat for the exit examination in cardiothoracic surgery. Only two candidates sat for the exit examination in cardiothoracic surgery of NPMCN in November 2015. This shows apathy towards a career in cardiothoracic surgery among junior surgical residents and a clear preference for other surgical specialities.

Cardiothoracic manpower is relatively unavailable in West Africa. In a study by Kesieme et al. in 2011, the number of active cardiothoracic surgeons serving a population of about 160 million people was about 26.^1^ Only 22% of cardiothoracic surgeons were active in cardiac surgery.^1^ The availability of cardiac surgeons per million of the population in North America and Europe is more than 10 times the figure for Africa.^2^ The cost of the diagnosis and treatment of heart disease is way beyond the means of the largely indigent population in Nigeria and West Africa.

Hence we carried out this study to rate the interest of junior surgical residents working in Nigeria in cardiothoracic surgery, weigh up their exposure in cardiothoracic surgery, ascertain factors they consider while making decisions on speciality choice, and examine factors that may be strong points or drawbacks in choosing a career in cardiothoracic surgery.

## Methods

We targeted all the junior surgical residents in the 40 institutions accredited either fully or partially by the faculty of surgery, NPMCN, between April and October 2015. An active trainer was identified in these institutions to help with data collection.

The data collection was done using the semi-structured questionnaire distributed to junior surgical residents who were doing rotations in the different surgical specialities in preparation for Part 1 of the examination of the Faculty of Surgery, NPMCN or WACS [postgraduate year (PGY) 1–3] or those who had completed their rotation but were yet to pass the examination (PGY > 3). We also used the opportunity provided by the Integrated Clinical and Revision Course in Surgery organised by WACS, between 6 and 9 September 2015 at Jos University Teaching Hospital, Jos, to collect data from some of our study population who we were unable to access.

A pilot study was carried out among 15 junior residents in one of the tertiary institutions to improve reliability of the research instruments. Additional inputs were provided by two active cardiothoracic surgeons and two senior residents in cardiothoracic surgery who were not part of the study.

The 56-item questionnaire was divided into four sections. The first section included socio-demographic data (age, gender, marital status, number of children and years spent in training). The second section included statements on the overall interest and exposure of the respondents in cardiothoracic surgery (CTS) as medical students and junior surgical residents and the influence of such rotation on their career. We also evaluated their active participation in major cardiothoracic procedures and the role of mentorship in their career. The relationship between the age of the respondents, their marital status, level of training, rotation in CTS in medical school and as a junior surgical resident and the different categories of interest in CTS was assessed.

The third section comprised statements evaluating the importance of the following factors in decision of speciality choice. These factors included type of procedures, job and research opportunities, opportunities to teach surgery, exposure to positive role models in the speciality, advice from colleagues, length of training and one that allows time for family.

In the fourth section, the respondents were asked to identify the shortcomings in practise of the different surgical specialities with regard to challenges in equipment, challenges in training, job dissatisfaction, income, unfriendly working atmosphere and time-consuming job. They were also asked to identify the factors that would most likely attract or reduce the interest of junior residents to CTS.

We retrieved the questionnaires from 238 respondents, accounting for a response rate of 61%. Quantitative statistics assessment was performed using SPSS 16.0 statistical software package (SPSS Inc; Chicago, IL). Categorical data were calculated in frequencies and percentages and the chi-squared test was used to test the level of significance. The level of statistical significance was kept at p < 0.05.

## Results

Out of 238 respondents who returned their questionnaires, 226 (95%) were males while 10 were females. The gender was not specified in two respondents. One hundred and thirty-seven respondents (57.6%) were in the age range between 31 and 35 years. The majority of respondents (66.8%) were in PGY-2 and more than half of them (52.1%) were married (Table 1).

**Table 1:** Demographic variables

*Demographics*	*Number*	*Percentage*
Age (years)		
26–30	59	24.8
31–35	137	57.6
36–40	35	14.7
41–45	7	2.9
Gender		
Male	226	95
Female	10	4.1
Not specified	2	0.9
Marital status	
Single	108	45.4
Married	124	52
Not specified	6	2.5
Number of children		
1	47	19.7
2	29	12.2
3	15	6.3
4	9	3.8
> 4	1	0.4
Level of training		
PGY-1	40	16.8
PGY-2	119	50
PGY-3	48	20.2
PGY < 3	31	13

Our respondents were grouped into four categories; 8.4% (n = 20) of the respondents were committed to specialising in CTS, 28.6% (n = 68) had not yet chosen a speciality but were interested in CTS, 38.7% (n = 92) had considered CTS at some point in their career but would choose another speciality, and 24.4% (n = 58) would neither choose nor were interested in CTS.

Among the cardiothoracic sub-specialities, the majority (37%) believed that paediatric cardiac surgery was the most exciting cardiothoracic sub-speciality. This was followed by adult cardiac surgery (21.8%) and general thoracic surgery (20.2%). A minority of respondents (8%) chose other cardiothoracic sub-specialities.

Out of the 20 respondents who were committed to specialising in CTS, 15% were in PGY-1, 50% were in PGY-2, 15% were in PGY-3, while 20% were in PGY > 3. Of all the 20 respondents who believed that paediatric cardiac surgery was the most exciting sub-speciality, 13 were committed to CTS.

The relationship between the different categories with regard to interest in CTS with level of training (p < 0.001) and the sub-speciality in CTS perceived as most exciting (p < 0.001) were statistically significant. Age, marital status and rotation in medical school did not have a relationship with interest in CTS (Table 2).

**Table 2:** The relationship between the four categories of interest in CTS with age, marital status, level of training, most exciting sub-speciality, and CTS rotation in medical school and as junior residents

	*A*	*B*	*C*	*D*	*p-value*
Age (years)					0.367
26–30	2	16	27	14	
31–35	13	38	53	33	
36–40	4	13	11	7	
41–45	1	1	1	4	
Marital status					0.361
Single	9	28	45	26	
Married	10	36	47	31	
Level of training					0.001
PGY-1	3	23	10	4	
PGY-2	10	33	51	25	
PGY-3	3	7	20	18	
PGY < 3	4	5	11	11	
Most exciting sub-speciality					0.001
Adult cardiac	5	23	20	4	
Paediatric cardiac	13	27	36	12	
General thoracic	1	15	23	9	
Other CT sub-specialities	1	3	4	-	
Not excited by any sub-speciality	-	-	9	33	
Have undertaken CT rotation as a junior resident					0.007
Yes	13	23	30	13	
No	7	45	62	45	
Undertook CT posting in medical school					0.176
Yes	13	33	36	28	
No	7	35	56	30	

A: committed to CTS; B: not yet chosen a speciality but interested in CTS; C: considered CTS but will choose another speciality; D: will neither choose nor interested in CTS.

More than half of the respondents (58%) had identified a mentor in surgery, of whom 7.6% were cardiothoracic surgeons. Those who were committed to CTS were more likely to have had a mentor than those who considered CTS but would choose another speciality (52 vs 44%) or those who would neither choose nor were interested in CTS.

One hundred and ten respondents (46.2%) undertook rotation as medical students in a cardiothoracic unit/s managing minor cardiothoracic cases. The average time spent in the rotation was two weeks in 73.1% of respondents. Among those who rotated in CTS, 72% believed that the rotation had a positive influence in their surgical career generally.

Only 33.2% of respondents (n = 79) had undertaken posting in CTS as junior surgical residents. In most instances, the posting

Only 33.2% of respondents (n = 79) had undertaken posting in CTS as junior surgical residents. In most instances, the posting lasted for three months. Most of the junior residents who were committed to undertaking a career in CTS underwent rotation in CTS in medical school and as junior surgical residents. The relationship between the different categories with regard to interest in CTS and rotation in CTS in medical school was however not statistically significant (p = 0.17), whereas that between the different categories and posting in CTS during junior surgical residency was statistically significant (p = 0.01) (Table 2).

In most instances, the cardiothoracic surgeons took an interest in the respondents during their posting. A major thoracic procedure had been observed by 50% (n = 119) of junior surgical residents, while only 13.4% (n = 32) had observed an open-heart surgical procedure. Only 28.2% (n = 67) and 2.1% (n = 5) had assisted in major thoracic procedures and open heart surgery, respectively.

## Assessment of factors that influence decision of speciality choice

**Fig. 1.**
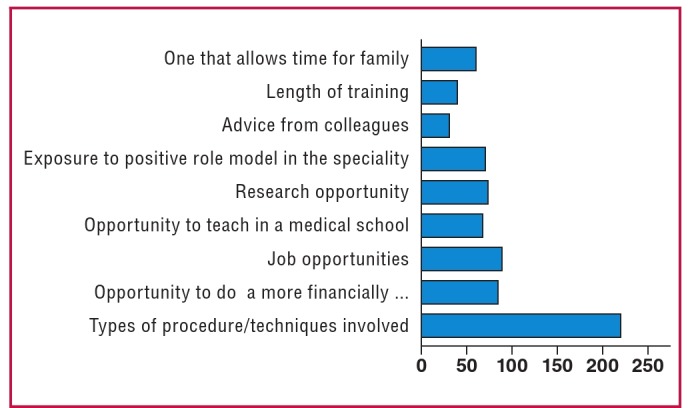
Graph showing factors considered most important in decision of surgical speciality choice.

Two hundred and nineteen respondents (92%) identified the type of procedure and the techniques involved in a speciality as the most significant factor considered in choice of surgical speciality. This was followed closely by job opportunities, which was identified by 89 respondents. Eighty-four respondents (35.3%) identified the opportunity to engage in a more financially rewarding practice as the third most popular factor (Fig. 1). The length of training and advice from colleagues were not popular factors.

The relationship between all factors considered in the decision of speciality choice and the different categories of interest in CTS among junior surgical residents in Nigeria were all statistically significant (p < 0.05). This is an indication that the variations across the categories of interest in CTS were not by chance.

**Fig. 2.**
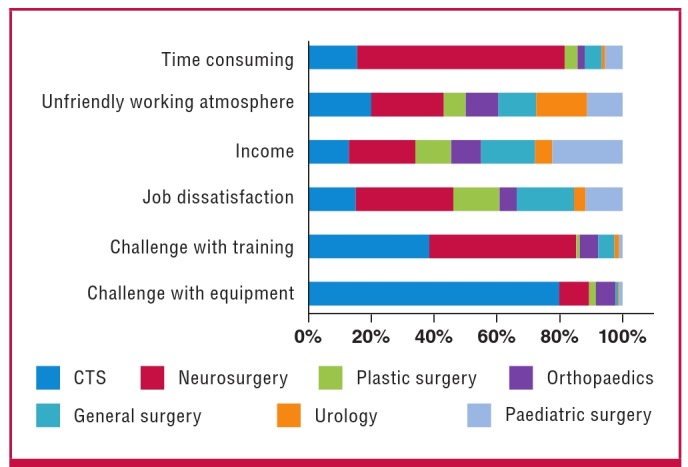
Graph showing shortcomings observed with the practice in different surgical specialties.

The respondents were asked about the most important shortcomings encountered in the practice of the following seven different surgical specialities (CTS, neurosurgery, plastic surgery, orthopaedics, general surgery, urology and paediatric surgery). One hundred and seventy-three respondents (72.7%) believed that the speciality with the greatest challenge with equipment was CTS. CTS was identified by 84 respondents (35.3%) to be second to neurosurgery in having the greatest challenge with training. Neurosurgery was believed to have the greatest shortcomings with regard to job dissatisfaction, unfriendly working atmosphere and being the most time consuming (Fig. 2).

## Factors affecting interest in CTS

**Fig. 3.**
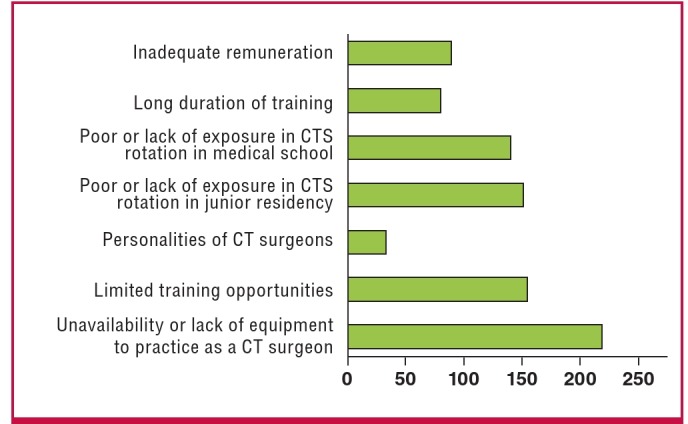
Graph showing the most important factors that junior surgical residents believe reduce interest in cardiothoracic specialisation.

Most of the respondents believed the most important factors that reduce the interest of junior residents in CTS were unavailability or lack of equipment to function as a cardiothoracic surgeon (91.2%), limited training positions (64.7%), poor or lack of exposure in CTS in medical school (58.8%) and poor or lack of exposure in CTS as junior surgical residents (63%) (Fig. 3). Among those who considered CTS but would specialise in another surgical discipline, 91.3% ticked unavailability of surgical equipment, accounting for the majority (38.7%) that identified this factor. This can be compared to those who were still to choose a surgical discipline but were interested in CTS(28.1%) and those who were already committed to specialising in CTS (7.8%). Those who considered CTS but would choose another surgical speciality also formed the majority of those who identified limited training positions (37% ), poor or lack of exposure in CTS as a junior resident (38%), and poor or lack of exposure in CTS in medical school (36%) as factors that reduced interest in CTS.

**Fig. 4.**
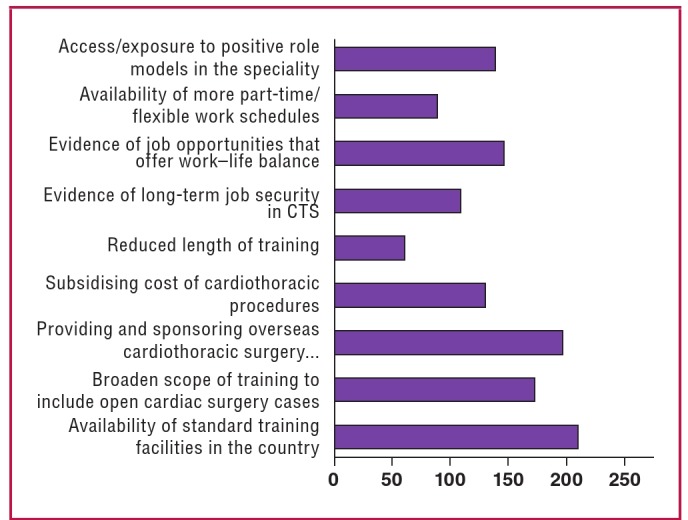
Graph showing the most important factors that would bias junior surgical residents towards cardiothoracic specialisation.

Two hundred and eight respondents (87.4%) believed that making more standard training facilities available in CTS in Nigeria would bias junior surgical residents towards pursuing a career in CTS. A large majority also believed that other important factors that could create a bias towards specialising in CTS were broadening the scope of training to include open cardiac cases (71.8%), providing and sponsoring cardiac surgery training and exposure abroad (81.9%), and providing evidence of job opportunities that would offer work–life balance (60.9%) (Fig. 4).

## Discussion

This study revealed a lack of interest among junior surgical residents in Nigeria towards pursing specialisation in CTS. A study done in the USA showed a declining interest among general surgery residents in choosing a career in CTS.^3^ This is similar to the experience in the UK where recent studies revealed a decline in interest in CTS among UK graduates.^4^,^5^ The entry point into a CTS programme in the West African sub-region is similar to that of the UK, where the application for entry is made at the level of PGY-2, unlike in the USA, where most cardiothoracic surgeons complete an initial general surgery training.

In Nigeria, no institution has been able to maintain and successfully sustain an open-heart surgery programme, despite a large number of cases requiring open-heart surgery, especially for rheumatic and congenital heart diseases.^6^-^8^ In 1974, open-heart surgery started at the University of Nigeria Teaching Hospital, Enugu (UNTH) and by 2002, about 102 surgeries had been recorded.^9^ The programme subsequently collapsed but fortunately it has recently been resuscitated by foreign medical missions.

Falase et al. reported 51 cases performed in Lagos State University Teaching Hospital, Ikeja (LASUTH) between August 2004 and December 2011, with the aid of a mission team. These cases were done despite gross challenges with support facilities and inadequately trained supportive staff.^10^ It is therefore understandable that only 13.4% of residents have observed cardiac surgery, and 2.1% have assisted in open-heart surgeries.

The failure of open-heart surgery programmes in Nigeria has largely been attributed to heavy financial outlay, intensive labour requirements and high resource consumption.^6^ In addition, the country has headed from oil boom to oil doom. However in nearby Ghana, the National Cardiothoracic Centre, Korle-Bu, reported 464 cases performed annually back in 2008, with 25% being open-heart procedures, especially surgeries for rheumatic and congenital heart diseases.^11^-^13^

Unavailability of equipment and lack of training positions are the key or primary factors that have reduced interest in CTS in Nigeria. These have ultimately led to the lack of adequately trained personnel and a fewer number of surgeons practising this speciality. This has resulted in secondary factors, such as poor or lack of exposure in CTS as surgical residents and as medical students. Only 32.2% of residents have therefore done a CTS posting. A higher percentage (46.2%) appeared to have undertaken rotations in CTS, as some units combine general surgery with CTS. Some cardiothoracic specialists also provide services for more than one institution. More residents are also likely to have received their undergraduate training in the larger institutions where there are cardiothoracic programmes.

The role of good exposure in CTS in medical school and in junior residency cannot be underestimated. A study by Lussiez et al. revealed that receiving adequate exposure in cardiothoracic procedures and disease management was significantly associated with higher satisfactory ratings in cardiothoracic procedures, especially thoracostomy incisions, empyema and pleural effusions, and lung cancer care.^14^ Good mentorship and absence of maltreatment were also positively correlated with good exposure.^14^ Good exposure in CTS in medical school may therefore play a role in biasing residents towards a career in CTS. For instance, Trehan et al. revealed that one-third of medical students who received a scholarship in CTS maintained their interest over time, and more than half maintained their interest in a surgical speciality.^15^

On account of these demographics and attitudes towards thoracic surgery, many foreign surgical societies have intensified efforts to attract medical and pre-medical students to a career in CTS. Cardiothoracic surgeons should identify and encourage those interested in CTS, and provide the necessary advice and mentorship.^16^

In the UK, the professional insecurity brought about by publication of surgeon-specific mortality data and the poor publicity surrounding cardiothoracic surgery is believed to be responsible for the decline in CTS among UK graduates.4 Bridgeman et al. also believed that relative lack of engagement with medical students is an additional factor.^17^

Vaporciyan et al. surveyed the factors affecting the interest in CTS among general surgery residents in the USA, and they noted that the dominant concern was job availability and security. Other important factors were mentorship and exposure to the CTS faculty.^3^ Lack of jobs, especially in the private sector, may have been responsible for the increased number of surgeons who sub-specialised in adult cardiac, paediatric cardiac, thoracic and transplantation surgery.^18^

The reasons for a decline in interest in CTS among junior surgical residents in Nigeria differed from those obtained from similar studies in developed countries. In the setting of a few thoracic surgeons in a densely populated country such as Nigeria, job availability is not likely to be an issue, as many university hospitals do not have a single thoracic surgeon.

## Conclusion

In Nigeria, CTS is unattractive to junior surgical residents. There is a need for government to improve sponsorship and provide facilities to commence, maintain and sustain open-heart surgery programmes. There is a need for cardiothoracic societiesin Africa to do more to provide opportunities to help and encourage medical students and junior residents. Cardiothoracic surgeons also need to identify and encourage those committed to pursuing a career in CTS and provide better mentorship to those who still have a chance of specialising in CTS.
